# Long-Term Impacts of the COVID-19 Pandemic on Drug/Alcohol Use Prevalence in a Population with Substance Use Disorders

**DOI:** 10.3390/ijerph20136261

**Published:** 2023-06-30

**Authors:** Alessio Gili, Massimo Lancia, Angela Gambelunghe, Luca Tomassini, Alessia Nicoletti, Kyriaki Aroni, Cristiana Gambelunghe

**Affiliations:** 1 Hygiene and Public Health Section, Department of Medicine and Surgery, University of Perugia, Piazza Lucio Severi, 06132 Perugia, Italy; alessio.gili@unipg.it; 2 Forensic Medicine, Forensic Science and Sports Medicine Section, Department of Medicine and Surgery, University of Perugia, Piazza Lucio Severi, 06132 Perugia, Italy; massimo.lancia@unipg.it (M.L.); luca.tomassini@unicam.it (L.T.); aroniky@libero.it (K.A.); 3 Occupational Medicine, Respiratory Diseases and Toxicology Section, Department of Medicine and Surgery, University of Perugia, Piazza Lucio Severi, 06132 Perugia, Italy; angela.gambelunghe@unipg.it

**Keywords:** COVID-19 pandemic, drug abuse, alcohol abuse, addiction, hair analysis

## Abstract

As time passes, the long-term effects of the COVID-19 pandemic are becoming increasingly apparent. The extreme restrictions imposed during the pandemic have had detrimental impacts on the most vulnerable groups, such as individuals suffering from substance and/or alcohol disorders (SUDs). This study reports quarterly laboratory data on alcohol and drug use in 150 subjects with SUDs that were examined using hair analysis for 2 years before the start of pandemic until after the end of the Italian health emergency. Overall, it was found that the number of subjects who used heroin, cocaine, and MDMA all decreased during the 2020 and 2021 lockdowns, increasing during reopening and subsequently stabilizing close to pre-COVID levels. Cannabis use was less impacted, remaining stable throughout the pandemic. Alcohol and benzodiazepine use both increased significantly during the lockdowns, displaying an opposing trend. While benzodiazepine use progressively returned to baseline levels, alcohol remained at significantly increased levels, even in September 2022. Long-term heavy drinking combined with substance use should be seriously considered, since these results in several health and social problems alongside alcohol-related comorbidities. Thus, appropriate response plans should be implemented both during and after the pandemic, whilst focusing on those who are most vulnerable.

## 1. Introduction

The coronavirus disease 2019 (COVID-19) pandemic has caused considerable changes in the daily lives of people around the world [1]. Many countries, during the first pandemic wave, were forced to introduce immediate emergency measures, including public lockdowns, self-quarantine, and social distancing, in an effort to mitigate the viral spread whilst reducing fatalities associated with the pandemic [1].

In Italy, a series of complex and stringent measures were in place for approximately 2 years aiming to contain the infectious disease. Beginning in March 2020, the first wave of the pandemic was accompanied by a “stay at home” strategy that involved shutting down all non-essential economic activities, such as educational institutions, retail outlets, and other businesses selling non-essential items [2]. These stringent emergency measures paid off, as new infections began to decline by late April 2020, with most social and business activities, including recreation, progressively resuming [3].

However, a second pandemic wave followed in the autumn of 2020, which prompted new and more severe restrictions (closing of public squares at 9 p.m.; curfew beginning at 10 p.m.; restaurant/bar/pub closures beginning at 6 p.m.; online schooling for 75% of secondary schools and universities; and closures of cinemas/theatres/music, halls/outdoors, and sports/activities) [4]. In November 2020, the Italian Government enforced containment for different scenarios based on local indicators of the epidemic risk known as a “zone/regional system” to avoid another nationwide lockdown [5].

Since the summer of 2021, the majority of social activities have resumed as a result of the intensive vaccination campaign. However, public participation was limited to those with a COVID-19 Green Certificate, which serves as proof that an individual has either had a COVID-19 vaccination, got a negative result from a virus test, or has recovered from COVID-19 [6].

From February 2022 onwards, ballrooms/discos/festivals have been definitively reopened, whilst the sanitary emergency ended on 31 March 2022, with the consequent progressive easing of all the remaining restrictive measures [7].

The prolonged social isolation during the pandemic affected all aspects of human life with many negative economic, social, and psychological effects, such as stress disorders, anxiety, depression, and substance abuse [8,9]. The impact of the COVID-19 pandemic on mental health cannot be underestimated, particularly concerning patients with substance and/or alcohol disorders (SUDs).

Heightened stress and anxiety levels alongside increasing isolation in a context of uncertainty may have affected some populations more than others. Due to the momentary absence of assistance from social and specialistic treatment services, people with SUDs were more likely to have been adversely affected by home confinement [10,11].

Difficulties in moving and the increased presence of security forces have substantially reduced classic drug trafficking on the streets. The greater difficulty in finding illegal substances has also resulted in an increase in drug prices [12]. Furthermore, the demand for recreational drugs, particularly cocaine and 3,4-methylenedioxymethamphetamine (MDMA), seems to have diminished in the short term, given the closure of non-essential recreational venues and the cancellation or postponement of festivals [13]. As a result, during the first pandemic wave, there was a shift toward substances that could be consumed in isolation and were more accessible [14,15,16,17].

Specific concerns were raised surrounding the misuse of benzodiazepines (BZDs), either diverted from therapeutic use or not licensed for medical use. Increased BZDs consumption during the pandemic was reported for various populations, including high-risk drug users, people in prison, and recreational drug users [13].

Although the results on changes in alcohol use patterns during lockdown were conflicting, there have been several reports primarily focused on web-based surveys of binge/heavy drinking during the lockdown alongside relapses post-lockdown [18,19]. Between April and July 2020, a cross-sectional online epidemiological study on 36,538 adults from 21 European countries discovered a general decline in alcohol use. This decrease was attributed to the reduction in heavy episodic consumption despite the fact there was a notable increase in alcohol consumption among those who had severe alcohol use [20].

Our preliminary data on drug and alcohol use patterns during the first wave of the pandemic in Italy based on toxicological findings from 30 subjects with SUDs monitored before, during, and immediately after the first 2020 lockdown demonstrated a proclivity to switch from illicit drugs to other potentially harmful but more accessible substances, such as alcohol and BZDs, with a corresponding decline in illicit drug use [21].

Through the present study conducted using a much larger number of subjects affected by SUDs (150) who were monitored continuously from December 2019 to September 2022 by hair analysis, we constructed a generalized estimating equation (GEE) model and reported alcohol/drug consumption in the long-term, including periods of lockdown and subsequent periods of reopening.

An extended study with 12 measurements for each subject at regular intervals allowed us to verify whether the changes induced during the most severe restrictions led to persistent changes, or whether there was a substantial return to drug/alcohol use habits before the pandemic. The GEE model produced more efficient estimates than conventional least square regression estimates in repeated studies. We believe that results from laboratory data presented here can contribute to improving our understanding of the long-term consequences of the pandemic on particularly vulnerable subjects.

## 2. Materials and Methods

### 2.1. Patient and Sample Collection

Laboratory procedures were carried out in accordance with the Declaration of Helsinki of 1975 (revised in 1983) and were approved by the Bioethics Review Board of the University of Perugia (Protocol 2012-006R). Informed consent was given by all participants; participation rate was near 90% for the number of patients undergoing the first toxicological check in December 2019. From December 2019 to September 2022, 3 cm of hair samples were collected from 150 participants every three months. All participants who provided consent had undergone complete analysis within the scheduled time. Participants were all from central Italy’s urban areas and were registered with the Public Service for Drug Dependence Treatment, where they underwent counseling therapy without the use of opiate substitution drugs, such as methadone or buprenorphine. The DSM-5 criteria were used to diagnose drug and/or alcohol disorders. The Forensic Toxicology Laboratory also conducted routine hair analyses on the subjects to check for drug and alcohol use. All patients provided prescription medication lists for other pathologies; however, BZDs were not part of the treatment plan.

### 2.2. Preparation and Analysis of Hair Samples

This study used previously described and fully validated methods [21]. Opiates (codeine, 6-acetylmorphine, and morphine), cocaine (and its metabolite benzoylecgonine), cannabinoids (D9-tetrahydrocannabinol and its metabolite 11-nor-9-carboxy-D9-tetrahydrocannabinol), amphetamine, methamphetamine, MDMA, benzodiazepines (BZDs: alprazolam, clonazepam, delorazepam, diazepam, flurazepam, lorazepam, midazolam, nitrazepam, oxazepam, temazepam, and triazolam), and cocaethylene (ethylbenzoylecgonine: the byproduct of concurrent consumption of alcohol and cocaine), were all analyzed using a triple quadrupole gas chromatography-tandem mass spectrometry (GC/MS-MS) 7000C GC/MS system (Agilent Technologies, Palo Alto, CA, USA) in selective ion monitoring mode.

To assess alcohol consumption using hair samples, ethyl β-D-6-glucuronide (EtG) was selected as a specific marker, and its extraction and analysis were performed using a fully validated method [22]. EtG chromatographic analysis was conducted using a 7890 B Agilent gas chromatograph (Agilent Technologies, Santa Clara, CA, USA) coupled to an Agilent 7000C triple quadruple mass spectrometer detector with an electron impact ion source. Data were acquired in multiple reaction monitoring modes. This method has also been fully validated and applied routinely in our laboratory [23].

### 2.3. Statistical Analysis

Descriptive statistics, including frequencies, percentages, frequency tables for categorical variables, and mean ± standard deviation (SD) for quantitative variables, were performed. Categorical variables were also analyzed and evaluated using chi-square analysis or Fisher’s exact test, as appropriate.

Additionally, analysis of variance for repeated measures was used to analyze changes in the use of drugs or alcohol every 3 months from December 2019 to September 2022, with results yielding a *p*-value < 0.05 being considered statistically significant.

To assess the evolution of consumption over time and to make inferences about the population when accounting for the within-subject correlation, a GEE model was constructed here. This method was an extension of generalized linear models to longitudinal or clustered data, where observations are no longer independent. This allowed more efficient estimates to be produced in repeated studies compared to ordinary least square regression since it accounted for correlations between observations. The model is also robust to the misspecification of correlation structure because the parameter estimates remain consistent. Moreover, these models use the available information from subjects that have missing measures, without imputing missing data (if the missingness is completely at random, MCAR) and, with difference as hierarchical models or random or mixed-effects models, the dependent variable can have a non-normal distribution and the predictor variables can be continuous, ordinal, or categorical.

Final models were evaluated, controlled by sex and age, considering 12 quarterly observations for each substance (cocaine, heroin, MDMA, cannabis, BZDs, and cocaethylene with a binary outcome across time within the same individual and EtG with a continuous outcome), and exchangeable correlation structures (all pairs of responses within a subject are equally correlated).

After model estimation, to better highlight the changes at each toxicological control, we used contrast approaches (with the delta method) using the reference data recorded before the start of the pandemic in December 2019.

A *p*-value of below 0.05 was considered to be statistically significant.

Statistical analysis was performed using STATA 14.1 (StataCorpLP, College Station, TX, USA) and R Statistical Computing Environment, “package geepack”.

## 3. Results

In this study, 150 subjects (aged 18–48 years, mean age: 28.72 ± 7.98; 77 females, 73 males) with SUDs were monitored retrospectively for alcohol and drug use before the pandemic and for every three months until September 2022, thus, obtaining 12 assessments for each subject. Because hair analyses enable the monitoring of relative changes in an individual using multiple samples taken over time, they were utilized in the present study [21]. A hair sample that was 3 cm long, allowed us to investigate a retrospective period of approximately 3 months before sampling, given that the average hair growth is 1 cm/month [24]. Therefore, results from the toxicological analyses carried out in December 2019 and March 2020 were considered as baseline levels that had not yet been affected by the restrictive measures implemented in response to the COVID-19 pandemic in March 2020. The statistical analysis of drug data was based on the decreases or increases observed in users measured at 12 intervals.

**Figure 1:**
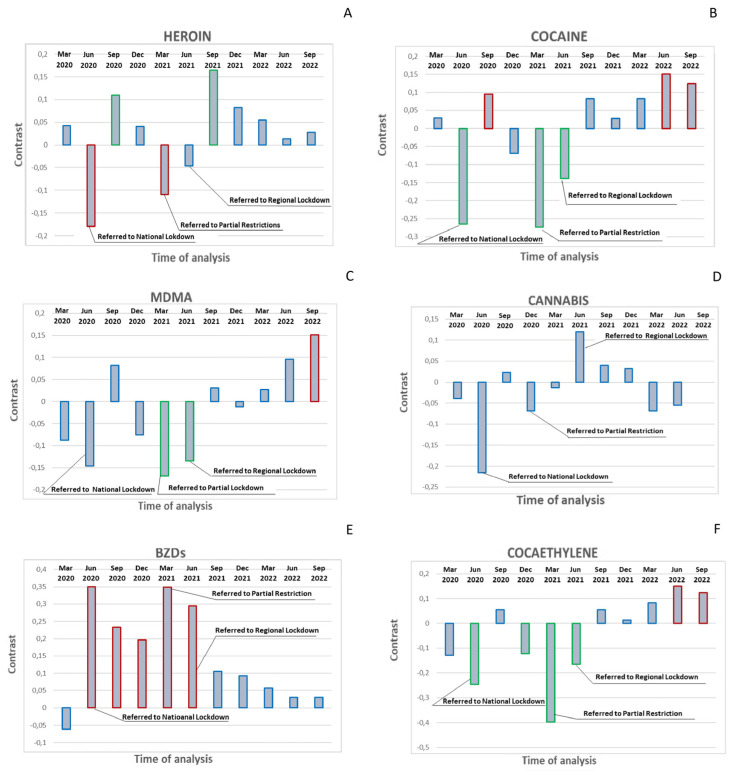
Contrast post-estimation after GEE model for heroin (**A**), cocaine (**B**), MDMA (**C**), cannabis (**D**), BZDs (**E**), and cocaethylene (**F**) at all monitoring times vs. baseline values in December 2019. Red outline = significantly higher than December 2019; green = significantly lower than December 2019; blue = not significantly different from December 2019.

The analysis of hair samples revealed a general significant decline in the use of illicit drugs during the 2020 and 2021 lockdown periods compared to that in the pre-lockdown controls. For example, the number of subjects who used heroin decreased significantly during the 2020 (contrast 0.18, *p* = 0.02) and 2021 (contrast 0.11, *p* = 0.02) lockdowns. However, significant increases were observed in September 2020 (contrast 0.11, *p* = 0.02) and September 2021 (contrast 0.16, *p* = 0.001); subsequently, heroin use progressively reduced before stabilizing at values similar to those observed pre-pandemic (Figure 1A and Table S1). In addition, decreases in the number of subjects testing positive for cocaine during the 2020 (contrast −0.26, *p* = 0.001) and 2021 (contrast −0.27, *p* < 0.001) lockdowns were observed. After the lockdown periods, cocaine-positive subjects increased to reach a statistical difference compared to baseline values in September 2020 (contrast 0.10, *p* = 0.043) and June 2022 (contrast 0,15, *p* = 0.001). This upward trend from the baseline was also observed until September 2022 (contrast 0.12, *p* = 0,009) (Figure 1B and Table S1). Furthermore, the number of samples that tested positive for MDMA decreased with no statistical difference during the 2020 lockdown, whereas a statistical difference (contrast −0.17, *p* = 0.001) was observed during the 2021 lockdown. The recovery from the consumption of this drug appeared to be slower than others, since positive subjects increased in September 2020 without a statistical difference, whilst at the end of the monitoring period, a statistical difference compared to baseline values was found (contrast 0.15, *p* = 0.004) (Figure 1C and Table S1). Additionally, cannabis consumption showed minor changes during the pandemic, since it did not decrease significantly during the 2020 lockdown. Moreover, in subsequent monitoring, there were no significant fluctuations in consumption until returning to baseline levels in September 2022 (Figure 1D and Table S1).

Notably, BZD consumption followed the inverse pattern of the traditionally abused drugs monitored in this study. The number of samples testing positive for BZDs increased significantly across a long period from June 2020 to June 2021, before declining gradually from September 2021 (Figure 1D, Table S1).

**Figure 2:**
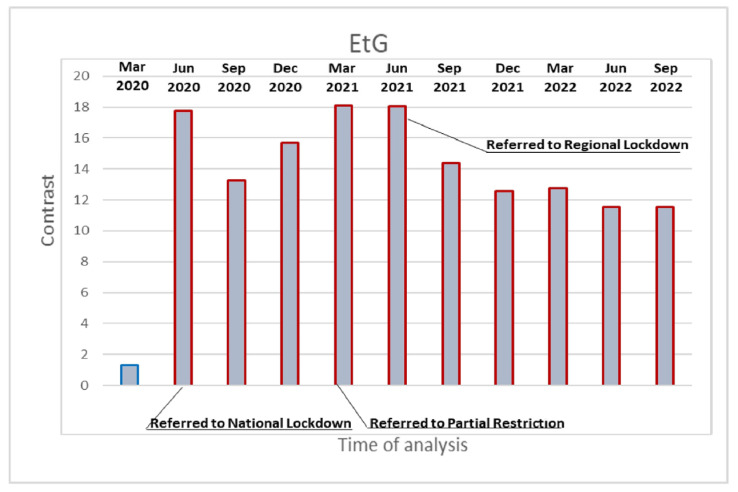
Contrast post-estimation after GEE model for EtG values measured in the hair at all monitoring times vs. baseline values in December 2019. Red outline = significantly higher than December 2019; blue = not significantly different from December 2019.

Alcohol intake showed a similar pattern to that for BZDs, which was monitored in the present study by measuring variations of EtG in hair, a helpful biomarker used to measure alcohol consumption during previous months [25]. Abstinence from alcohol could be demonstrated using internationally adopted cut-off concentrations (EtG in hair < 5 pg/mg), whereas chronic excessive drinking with a daily ethanol consumption of 60 g or more could also be determined (EtG in hair > 30 pg/mg) [23,26]. Statistical analysis of alcohol data was based on the amount of EtG measured in the hair of the participants over 12 three-month intervals. The EtG values detected throughout 2 years of monitoring were significantly higher compared to pre-lockdown levels (EtG median: 41.65 ± 13.96 and 43.13 ± 15.00), with the highest values being recorded after the 2020 lockdowns (EtG median: 59.50 ± 16.07; contrast 17,768, *p* < 0.001) and 2021 (EtG median: 57.41 ± 17.58; contrast 18.05, *p* < 0.001). Notably, even in the final monitoring periods, when the restrictive measures had definitively ended in Italy, they remained significantly higher than the baselines in September 2022 (EtG median value 51.58 ± 24.36; contrast 11.548 *p* < 0.001) (Figure 2, Table S1). Furthermore, 80% of the subjects had an EtG value > 30 pg/mg before lockdown, with 100% and 96% during the 2020 and 2021 lockdowns, respectively, with 75% at the last control. According to the measurement of cocaethylene (Figure 1F and Table S1), the metabolite produced when cocaine and ethanol are consumed together, almost all subjects who used cocaine also drank alcohol at the same time (85% before lockdown, 100% and 94% during the 2020 and 2021 lockdowns, respectively, and 100% at the final control).

## 4. Discussion

The COVID-19 pandemic had an impact on every aspect of people’s lives, leading to psychological disorders, alteration in health-related behaviors, and addiction-related problems, including drug/alcohol use [27]. Many indicators have highlighted the adverse effects of the pandemic on the choice of substances used by people, the frequency with which these substances are used, and the number of people requiring subsequent treatment [28].

The current study’s data revealed a general decrease in illicit drug use during the lockdowns of 2020 and 2021, while usage of alcohol and BZDs both considerably increased. Although BZD use had gradually returned to pre-pandemic levels, alcohol use increased significantly even in September 2022.

In this regard, European data recorded to date have been conflicting. According to an analysis of household sales data, more alcohol was purchased during lockdown in the United Kingdom. Most studies have relied on surveys. One study in France indicated self-reported increases in alcohol and tobacco consumption but no general change in cannabis use [29]; another previous study performed in Greece showed a reduction in alcohol and cannabis use alongside an increase in nicotine use [30].

However, few studies have been based on laboratory analysis data. Results from wastewater analysis in seven cities in the Netherlands, Belgium, and Italy indicated a heterogeneous impact from the first wave of COVID-19 on illicit drug use [31]. In fact, in some places, consumption remained unchanged or increased compared to the control period of the previous year, whilst there were increases elsewhere [31]. One study conducted in Turkey on a large number of suspected drug users through blood or urine tests showed a significant decrease in the use of classic illicit drugs [32].

Preliminary data from our previous study, based on hair analysis in 30 subjects with SUDs, showed that whereas BZD and alcohol consumption increased during the first wave of the COVID-19 pandemic in Italy, illicit drug use decreased overall [21]. Through the present study on the same type of subjects, but in larger numbers (150, equally represented by males and females) for 2 years throughout the pandemic, considering the periods of lockdown and reopening, we constructed a GEE model to verify the highlighted trend, while uncovering new information.

First, even if the 2021 Italian lockdown had less restrictive measures than the first (work activities, public offices, and shops were all open), it produced a similar effect on the consumption of alcohol and drugs, probably since the decrease was primarily due to the curfew at night, the closure of recreational activities, and the restrictions of movement outside the municipality of residence.

With the exception of cannabis, the decline in the number of subjects who used heroin, cocaine, and MDMA occurred in both the 2020 and 2021 lockdowns, with a general recovery in use being observed during the reopening phase, which corresponded to the summer of 2020 and 2021. From September 2021, the number of heroin users returned to baseline values, while cocaine and MDMA users remained at an increased level, even at the final monitoring interval. These data were consistent with the shortages of heroin in 2020 reported by several countries, suggesting that transport limitations imposed by the COVID-19 pandemic may have blocked trafficking along the Balkan route into the European Union. However, from 2021, heroin availability returned to pre-pandemic levels [33]. Additionally, the reduction in cocaine and MDMA use during COVID-19 restrictions may have been caused by the cessation of nightlife and entertainment venues, which are typically related to the use of these drugs [33]. Although more recent data from various sources have suggested cocaine’s availability, its use remains very high in comparison to historical standards [33].

According to data from wastewater MDMA residue analysis, drug-checking services, and focus groups with service providers, MDMA use was still below pre-pandemic levels during 2021 [33]. In Italy, the reopening of concert halls and dance venues took place only in February 2022; in fact, it was only during the following monitoring periods that significant increases in MDMA consumers were found compared to the baseline.

The number of subjects who used cannabis was not substantially affected by the pandemic. Cannabis use did not decrease considerably during the lockdown in 2020, whilst showing minor fluctuations during the reopening periods, before stabilizing in September 2022 at values similar to the baseline. Unlike other drugs, many studies have suggested that cannabis use was relatively unaffected by social distancing measures and stay-at-home orders [34,35]. Throughout the pandemic, cannabis cultivation levels in European countries maintained largely unchanged; therefore, its availability returned to normal levels shortly after the first lockdown, even when European countries reintroduced new restrictive social distancing measures [34].

Notably, variations in the use of BZDs showed an inverse trend compared to that of the other monitored substances. The usage of BZDs constituted drug misuse in all respects as they were not prescribed to the study subjects’ patients. The increase in the number of BZD users occurred during periods of immense social isolation, during which the use of other drugs decreased; from June 2021, the number of BZD consumers gradually returned to baseline values when the consumption of other illicit drugs also began to grow.

The increased usage of BZDs could be attributed to their easy availability and low price alongside pandemic-related mental health issues. A sample of sentinel hospitals reported a rise in BZD-related emergency cases in 2020 compared with those in 2019 [34]. Furthermore, many studies conducted in different countries confirmed the significant misuse and abuse of BZDs during the pandemic [17]. BZDs, whose therapeutic use is approved for the treatment of insomnia, anxiety, epilepsy, and panic disorder, have a high potential for misuse and are frequently combined when abused by people with SUDs, with opioids and/or alcohol constituting the most frequent primary substances [23]. BZDs are often misused to boost the euphoric effects of other drugs and to mitigate adverse effects, such as insomnia caused by stimulant use, as well as to reduce withdrawal symptoms between doses [21]. In the most vulnerable groups, such as individuals with SUDs, restrictive social measures may have increased loneliness, anxiety, anger, and sleep disorders, resulting in the increased use of self-care and easily accessible substances, such as BZDs.

In this study, the pattern of increased BZD use was also observed for alcohol. Changes in alcohol use during lockdown have been reported in the European Monitoring Centre for Drugs and Drug Addiction Trendspotter briefings [14], which included more frequent drinking, drinking in greater quantities, and drinking alone.

More recent studies have also shown that this behavior was typical of people with SUDs rather than the general population, which severely limited social drinking due to restrictions [11,20]. Vulnerable subjects, who were isolated at home without access to social or medical help and were often unemployed, with a pre-existing attitude to hard drinking significantly increased their alcohol consumption [11]. It is, however, important to note that unlike the other substances monitored, whose fluctuations appear to have been closely linked to restrictive measures, alcohol consumption displayed a different trend. Despite a slight decrease since June 2021, this remained dangerously high for all measures, never returning to baseline. Notably, at the final monitoring period, the number of subjects consuming alcohol above the threshold values was lower than that of the other controls, whilst the number of people abusing alcohol increased. Therefore, the increase in alcohol consumption during the lockdown stabilized, with the initial conditions having worsened in the examined subjects.

The determination of cocaethylene, a metabolite formed by the liver when cocaine and alcohol are consumed together, indicated that most cocaine users had simultaneously abused alcohol during both years of monitoring. High levels of alcohol consumption over two years with the combined consumption of drugs are of great concern for increased risk of morbidity and mortality, social, and legal problems [36]. Acute intoxication can result in risky sexual behavior, workplace accidents, and episodes of violence and drunk driving, which are responsible for a large percentage of fatal road accidents [36].

Although the present research was limited to people who were at an elevated risk of using alcohol and/or drugs and, thus, the results are not representative of the general population, the strength lies in multiple longitudinal measurements. Through these measurements, we can better understand the overall impact of the pandemic and its long-term consequences on this vulnerable group.

Implementing specific interventions after the pandemic is crucial for epidemiological monitoring, health and social planning, and combating the hazardous and detrimental consumption of alcohol among the general population, particularly in drug/alcohol high-risk groups.

## 5. Conclusions

The long-lasting consequences of the COVID-19 pandemic impact not only the physical and psychological health of individuals, but also the community’s well-being and the health service’s resilience. Data obtained showed a change in drug/alcohol use patterns during the pandemic. While changes in drug use were closely related to stringent restrictive measures during the 2020 and 2021 lockdowns and subsequent phases of reopening, a lack of control about drinking was observed. Although the pandemic had an adverse effect on almost everyone in society, policies must pay specific attention to the long-term impacts on the most vulnerable groups. A careful assessment of the population at risk (alcohol/drug users, users of treatment services, etc.) is essential to analyze the risk of alcohol while obtaining useful information for the planning of health policies and the evaluation of necessary interventions (Health emergency and preventive measures, availability of health services, systems for rapid identification of risks, diagnosis, and treatment).

## Data Availability

The data presented in this study are available on request from the corresponding author. The data are not publicly available due to privacy restrictions.

## Supplementary Materials

The following supporting information can be downloaded at: https://www.mdpi.com/article/10.3390/ijerph20136261/s1, Table S1: Results from Generalized Estimating Equations models for each substance analyzed.
