# Supplementary material for: Long-Term Impacts of the COVID-19 Pandemic on Drug/Alcohol Use Prevalence in a Population with Substance Use Disorders
Source: Int J Environ Res Public Health. 2023 Jun 30;20(13):6261. doi: 10.3390/ijerph20136261 (PMC10341792; doi:10.3390/ijerph20136261)
Supplement: Supplementary file 1 [file ijerph-20-06261-s001.zip › ijerph-2346667-supplementary.pdf]

Heroin						
Time	Contrast	Std. Err.	z	P>z	[95% Conf.	Interval]
Mar20 vs Dec19	0,04	0,08	0,55	0,58	-0,11	0,20
Jun20 vs Dec19	-0,18	0,08	-2,31	0,02	-0,33	-0,03
Sep20 vs Dec19	0,11	0,05	2,31	0,02	0,02	0,20
Dec20 vs Dec19	0,04	0,05	0,87	0,39	-0,05	0,13
Mar21 vs Dec19	-0,11	0,05	-2,31	0,02	-0,20	-0,02
Jun21 vs Dec19	-0,05	0,05	-0,94	0,35	-0,14	0,05
Sep21 vs Dec19	0,16	0,05	3,46	0,00	0,07	0,26
Dec21 vs Dec19	0,08	0,05	1,73	0,08	-0,01	0,18
Mar22 vs Dec19	0,05	0,05	1,15	0,25	-0,04	0,15
Jun22 vs Dec19	0,01	0,05	0,29	0,77	-0,08	0,11
Sep22 vs Dec19	0,03	0,05	0,58	0,56	-0,07	0,12

BZDs						
Time	Contrast	Std. Err.	z	P>z	[95% Conf.	Interval]
Mar20 vs Dec19	-0,06	0,12	-0,50	0,62	-0,30	0,18
Jun20 vs Dec19	0,35	0,12	2,85	0,00	0,11	0,59
Sep20 vs Dec19	0,23	0,07	3,12	0,00	0,09	0,38
Dec20 vs Dec19	0,20	0,07	2,64	0,01	0,05	0,34
Mar21 vs Dec19	0,35	0,07	4,69	0,00	0,20	0,49
Jun21 vs Dec19	0,29	0,07	3,93	0,00	0,15	0,44
Sep21 vs Dec19	0,11	0,07	1,41	0,16	-0,04	0,25
Dec21 vs Dec19	0,09	0,07	1,24	0,22	-0,05	0,24
Mar22 vs Dec19	0,06	0,07	0,77	0,44	-0,09	0,20
Jun22 vs Dec19	0,03	0,07	0,40	0,69	-0,12	0,18
Sep22 vs Dec19	0,03	0,07	0,40	0,69	-0,12	0,18

Cocaine						
Time	Contrast	Std. Err.	z	P>z	[95% Conf.	Interval]
Mar20 vs Dec19	0,03	0,08	0,37	0,71	-0,13	0,18
Jun20 vs Dec19	-0,26	0,08	-3,34	0,00	-0,42	-0,11
Sep20 vs Dec19	0,10	0,05	2,02	0,04	0,00	0,19
Dec20 vs Dec19	-0,07	0,05	-1,44	0,15	-0,16	0,02
Mar21 vs Dec19	-0,27	0,05	-5,78	0,00	-0,37	-0,18
Jun21 vs Dec19	-0,14	0,05	-2,91	0,00	-0,23	-0,05
Sep21 vs Dec19	0,08	0,05	1,73	0,08	-0,01	0,18
Dec21 vs Dec19	0,03	0,05	0,58	0,56	-0,07	0,12
Mar22 vs Dec19	0,08	0,05	1,73	0,08	-0,01	0,18
Jun22 vs Dec19	0,15	0,05	3,18	0,00	0,06	0,24
Sep22 vs Dec19	0,12	0,05	2,60	0,01	0,03	0,22

MDMA						
Time	Contrast	Std. Err.	z	P>z	[95% Conf.	Interval]
Mar20 vs Dec19	-0,09	0,09	-1,00	0,32	-0,26	0,08
Jun20 vs Dec19	-0,15	0,09	-1,68	0,09	-0,32	0,02
Sep20 vs Dec19	0,08	0,05	1,58	0,11	-0,02	0,18
Dec20 vs Dec19	-0,08	0,05	-1,45	0,15	-0,18	0,03
Mar21 vs Dec19	-0,17	0,05	-3,21	0,00	-0,27	-0,07
Jun21 vs Dec19	-0,13	0,05	-2,56	0,01	-0,24	-0,03
Sep21 vs Dec19	0,03	0,05	0,59	0,56	-0,07	0,13
Dec21 vs Dec19	-0,01	0,05	-0,23	0,82	-0,11	0,09
Mar22 vs Dec19	0,03	0,05	0,53	0,60	-0,07	0,13
Jun22 vs Dec19	0,10	0,05	1,84	0,07	-0,01	0,20
Sep22 vs Dec19	0,15	0,05	2,89	0,00	0,05	0,25

Cannabis						
Time	Contrast	Std. Err.	z	P>z	[95% Conf.	Interval]
Mar20 vs Dec19	-0,04	0,12	-0,34	0,74	-0,27	0,19
Jun20 vs Dec19	-0,22	0,12	-1,86	0,06	-0,44	0,01
Sep20 vs Dec19	0,02	0,07	0,33	0,74	-0,11	0,16
Dec20 vs Dec19	-0,07	0,07	-0,98	0,33	-0,20	0,07
Mar21 vs Dec19	-0,01	0,07	-0,20	0,84	-0,15	0,12
Jun21 vs Dec19	0,12	0,07	1,70	0,09	-0,02	0,26
Sep21 vs Dec19	0,04	0,07	0,57	0,57	-0,10	0,18
Dec21 vs Dec19	0,03	0,07	0,46	0,65	-0,10	0,17
Mar22 vs Dec19	-0,07	0,07	-0,98	0,33	-0,20	0,07
Jun22 vs Dec19	-0,05	0,07	-0,79	0,43	-0,19	0,08
Sep22 vs Dec19	0,00	0,07	0,00	1,00	-0,14	0,14

Cocaethylene						
Time	Contrast	Std. Err.	z	P>z	[95% Conf.	Interval]
Mar20 vs Dec19	-0,13	0,09	-1,47	0,14	-0,30	0,04
Jun20 vs Dec19	-0,25	0,09	-2,82	0,01	-0,42	-0,08
Sep20 vs Dec19	0,05	0,05	1,05	0,29	-0,05	0,16
Dec20 vs Dec19	-0,12	0,05	-2,36	0,02	-0,23	-0,02
Mar21 vs Dec19	-0,40	0,05	-7,61	0,00	-0,50	-0,29
Jun21 vs Dec19	-0,17	0,05	-3,16	0,00	-0,27	-0,06
Sep21 vs Dec19	0,05	0,05	1,05	0,29	-0,05	0,16
Dec21 vs Dec19	0,01	0,05	0,26	0,79	-0,09	0,12
Mar22 vs Dec19	0,08	0,05	1,57	0,12	-0,02	0,18
Jun22 vs Dec19	0,15	0,05	2,88	0,00	0,05	0,25
Sep22 vs Dec19	0,12	0,05	2,36	0,02	0,02	0,23

EtG						
Time	Contrast	Std. Err.	z	P>z	[95% Conf.	Interval]
Mar20 vs Dec19	1,30	2,75	0,47	0,64	-4,08	6,68
Jun20 vs Dec19	17,77	2,75	6,47	0,00	12,39	23,15
Sep20 vs Dec19	13,26	1,64	8,09	0,00	10,05	16,47
Dec20 vs Dec19	15,70	1,64	9,58	0,00	12,49	18,91
Mar21 vs Dec19	18,08	1,64	11,03	0,00	14,87	21,30
Jun21 vs Dec19	18,05	1,65	10,92	0,00	14,81	21,28
Sep21 vs Dec19	14,40	1,64	8,78	0,00	11,18	17,61
Dec21 vs Dec19	12,55	1,64	7,65	0,00	9,34	15,76
Mar22 vs Dec19	12,75	1,64	7,78	0,00	9,54	15,97
Jun22 vs Dec19	11,55	1,64	7,04	0,00	8,34	14,76
Sep22 vs Dec19	11,55	1,64	7,04	0,00	8,34	14,76
